# Supplementary material for: Chromothripsis during telomere crisis is independent of NHEJ, and consistent with a replicative origin
Source: Genome Res. 2019 May;29(5):737–49. doi: 10.1101/gr.240705.118 (PMC6499312; doi:10.1101/gr.240705.118)
Supplement: Supplemental Material [file supp_gr.240705.118_Supplemental_file_1.zip › contigs/annotated_contigs/DB116/contig.2.DB116_length_610_mean_cov_9.09672131148.docx]

**DB116_length_610_mean_cov_9.09672131148**

```
ATACAGAGTGTCTGGCCAATATGTGCTCAATAAATAGGTATTAAATTCAGTTGAATTAAAAGAAAATTGTATGTTCCTTATTTAATTCA
 >chr11:80571995-80572353 + E=9e-204
TTTTATTCAAGTATATCCCATTTTATAATTTTAAGGAACTTAATCGATTTAAAGAAGTAGATTAATCTGTCCAAGGTTGTCAAGTAATA

ACTGGAACAATCAGAATTAGAATGCACAAAGAACAAAGGAAAAGATATTTAAAGGGAGGTACTGTAACAACTACCAAGTCAGAAAGATT

CTTGAGAATGTAGATGATATAATGTATTTTTGTCCCTGTAAATAGTGAATAACAATAAATTAATAAACATTTGACAAATAATAAATCA|

TAC|AGGCACCCACCACCACACCCAGCTAAATTTTTTTGTATTTTTAGTAGAGATGGGGTTTCACCATGTTAGCCAGGCTGGTCTTGAA
>chr11:80589742-80590003 + E=2e-131
TTCCTGACCTCAGGTGATCTGCCTGCCTTGGCCTCCCAAACTGCTACAGGCGTGAGCCACCGTGCCCAGACAAAAGCCTTTCATCTAGC

TATTTTCTAATATATAATACCTTATTGTTAACCACCTTTGCAAAGGTTATACTGTCATATTTCCTCTTCTAGCCCTTT
```
